# The Impact of Gut Microbiota on Post-Stroke Management

**DOI:** 10.3389/fcimb.2021.724376

**Published:** 2021-10-12

**Authors:** Junyi Zhao, Siyu Liu, Jingyi Yan, Xinzhou Zhu

**Affiliations:** ^1^ The Brain Cognition and Brain Disease Institute (BCBDI), Shenzhen Institute of Advanced Technology, Chinese Academy of Sciences, Shenzhen, China; ^2^ Shenzhen-Hong Kong Institute of Brain Science-Shenzhen Fundamental Research Institutions, Shenzhen, China; ^3^ Department of Laboratory Medicine, Karolinska Institute, Stockholm, Sweden

**Keywords:** gut microbiota, schemic stroke, immune response, body temperature, blood glucose, blood pressure, oxygen, hydration

## Introduction

According to the recent Global Burden of Disease (GBD) study, stroke is the leading cause of death and disability, particularly in aged population (Collaborators, 2019). In the last decades, the mortality rate of stroke has significantly decreased, and the disability-adjusted life year (DALY) and years lived with disability (YLD) have been controlled (Collaborators, 2019). Nevertheless, stroke remains as a major health concern in both developed and developing countries (Collaborators, 2019). Novel therapeutic methods and improved management in stroke prevention and post-stroke recovery are still urgently demanded.

In a long period, most studies focused on the cardiovascular and neurological aspects of stroke, while only a small group of researchers kept an eye on the pathological alterations in gastrointestinal tract of stroke patients (Schaller et al., 2006). These studies mainly discussed the consequences of impaired nutritional status in stroke events (Schaller et al., 2006). Notably, in the past ten years, the ecosystem of microbiota in gastrointestinal tract has been linked to various physiological and pathological processes (Donaldson et al., 2016). Increasing evidences have demonstrated that the compositional changes of gut microbiota complexity are involved in diverse gastrointestinal disorders (Manichanh et al., 2006) and metabolic dysfunctions such as obesity and diabetes (Wen et al., 2008; Ridaura et al., 2013), which may also contribute to the nutrition status after stroke. Moreover, gut microbiota is recently considered to communicate with central nervous system in a bidirectional pattern (Collins et al., 2012). The metabolic products of gut microbiota regulate not only normal brain development but also various brain disorders through neural, immunological, endocrinal and metabolic pathways (Collins et al., 2012). Therefore, deep insights into the relationship between gut microbiota and stroke could provide novel avenues to improve post-stroke recovery and prevent stroke recurrence.

More than 85% of stroke events are caused by the blockage of blood flow, namely ischemic stroke (Moskowitz et al., 2010), thus we focused on ischemic stroke in this review. We summarized recent advances in the interactions between commensal gut microbiota and ischemic stroke: how stroke insult changes gut microbiota composition and how these shifts reversely influence stroke outcome and prognosis. We also attempted to figure out the clues from latest literatures by which gut microbiota may affect the major aspects during post-stroke management, including the controls of body temperature, blood glucose, blood pressure, oxygen and hydration (Bhalla et al., 2001). The concerns on gut microbiota will provide researchers novel therapeutic potentials for ischemic stroke and remind clinicians for special cares in post-stroke management.

## Literature Search Strategy

We used PubMed and Google Scholar to search recent advances in the relationship between gut microbiota and ischemic stroke. “Gut microbiome” and “intestinal flora” were used as synonyms of “gut microbiota”. As for the progresses in the main aspects of post-stroke management, key words including “immune response”, “inflammation”, “body temperature”, “hypothermia”, “hyperthermia”, “blood glucose”, “hypoglycermia”, “hyperglycermia”, “blood pressure”, “hypotension”, “hypertension”, “oxygen”, “hypoxia”, “hyperoxia”, “hydration”, “dehydration” and “overhydration” were combined with “gut microbiota” and its synonyms to search related references.

Studies in the field of gut microbiota-stroke relationship burst mainly in 2016, therefore we focused on the research articles in this year and afterwards. Review articles were only included when they provided novel insights in this area. Background knowledge may be referred to high-quality research articles or review articles before 2016. In a total of >200 references were included in the first round of literature search. After removing duplicating information or unsolid studies, eventually 86 references were selected and cited.

## Gut Microbiota and Brain Disorders

In a healthy human, over 100 trillion microorganisms reside predominantly in gastrointestinal tract (Qin et al., 2010). Among gut microbial community, *Bacteroidetes* and *Firmicutes* are two main phylotypes which constitute more than 90% of the core microbiome shared by all individuals, while *Actinobacteria*, *Proteobacteria* and *Verrucomicrobia* dominate the remaining part (Qin et al., 2010). Interestingly, in murine gastrointestinal tract, the composition of microbiome is of high similarity as that in human, with *Bacteroidetes* and *Firmicutes* as the most dominant phyla (>90%), and *Proteobacteria*, *Cyanobacteria*, *Tenericutes*, *Actinobacteria* and *Deferribacteres* largely occupying the rest proportion (Cho et al., 2012; Gu et al., 2013). Although the core microbiome between mouse and human gut share a high qualitative similarity, the species are more variable at lower taxonomic level. In addition, the abundance of specific phyla and species is also observed to be different between mouse and human (Krych et al., 2013). Therefore, it remains challenging to establish a humanized gnotobiotic mouse model which authentically represents the compositional and metabolic alterations of human gut microbiota after intervention. Many careful considerations are required in model designing, including the isolation, storage and transplantation conditions of human feces, the genetic background of mouse, and the diet ingredients of both mouse and human (Park and Im, 2020).

The concept of gut-brain axis (GBA) has been established for a long history. GBA consists of bidirectional communications between gastrointestinal digestive functions and brain activities, and gut microbiota interacts with GBA in a complex pattern involving autonomic, endocrinal and immune crosstalk (Cryan and Dinan, 2012). On one hand, cerebral neuroendocrinal changes triggers the homeostasis of gut microbiota through hypothalamic-pituitary-adrenal (HPA) axis; on the other hand, gut microbiota conversely coordinates brain functions through their metabolic products and the modulation of immune cells (Fung et al., 2017). In the past decade, massive studies have unraveled diverse roles of gut microbiota not only in normal brain development (Diaz Heijtz et al., 2011), but also in cerebral pathological conditions, including acute brain injuries, chronic neurodegeneration and mood disorders (Houlden et al., 2016; Wu et al., 2017; Zheng et al., 2019).

## The Interplay Between Stroke and Gut Microbiota

Emerging evidence have revealed that the dysbiosis of gut microbiota can be induced by ischemic stroke in both rodents and patients. Ischemic stroke can cause massive goblet cell and enteric nerve loss, breakdown of mucus layer and disruption of gut barrier, leading to subsequent dysbiosis and translocation of gut microbiota (Durgan et al., 2019). In a mouse model of ischemic stroke, the injury showed a remarkable impact on reshaping gut microbiota population, including the most abundant phylotypes *Firmicutes*, *Bacteroidetes*, and *Actinobacteria* as mentioned above (Singh et al., 2016). The species diversity of gut microbiota was also reduced upon injury (Singh et al., 2016). In another model of pig stroke, the abundance of the *Proteobacteria* dramatically increased after stroke, while *Firmicutes* and *Lactobacillus* decreased accordingly (Jeon et al., 2020). In clinical studies, intestinal dysbiosis was consistently observed in patients with acute ischemic stroke, when comparing with healthy control group (Xia et al., 2019; Xu et al., 2021). Among the major gut microbiota populations, *Parabacteroides*, *Oscillospira* and Enterobacteriaceae were enriched in stroke patients, while *Prevotella*, *Roseburia* and *fecalibacterium* were in contrast reduced (Xia et al., 2019). Notably, in an early stage of stroke recovery, Enterobacteriaceae enrichment was observed to highly correlate with high risk and poor outcome, making it as a potential biomarker of ischemic stroke (Xu et al., 2021). Moreover, the detrimental effects of post-stroke dysbiosis are not restricted in gastrointestinal tract, but also in other organs as a consequence of translocation and dissemination. For instance, typical intestinal bacterial species such as *Enterococcus* spp., *Escherichia coli* and *Morganella morganii* can translocate into lung and cause severe infection after ischemic injury (Stanley et al., 2016).

On the other hand, dysregulated compositions of gut microbiota can in turn influence stroke outcomes. When stroke induces dysbiosis of gut microbiota, Enterobacteriaceae in gut microbiota can also accelerate systematic inflammation thus exacerbate brain damage in both mouse model and patients samples, which may serve as a promising therapeutic target (Xu et al., 2021). In another animal study, atorvastatin restored gut microbiota homeostasis, contributing to its anti-inflammatory functions after stroke (Zhang et al., 2021).

Based on the evidence in both animal models and clinical studies, the alterations of gut microbiota phyla are believed to present a strong correlation with ischemic stroke, thus may be used as indicators of stroke incidence, progress and prognosis (**Table 1**). In addition, dysbiosis and infections in other major organs should be carefully considered and prevented in stroke patients. Meanwhile, stroke-induced dysbiosis of gut microbiota can also exacerbates brain injury and negatively influence stroke outcome, which may serve as novel biomarkers and therapeutic targets of stroke (**Table 1**).

**Table 1:** Evidences of the correlation between gut microbiota and ischemic stroke.

Animal model or clinical study	Key findings and potential therapeutic strategies	References
Mouse MCAO	Gut micriobota dysbiosis induces pro-inflammatory T cells in gut and ischemic brain, which may serve as a target to reduce ischemic infaction.	(Singh et al., 2016)
Mouse MCAO	IL-17+ γδ T can be used as an immunomodulatory target to restore gut microbiota and promote stroke recovery.	(Benakis et al., 2016)
Mouse MCAO	Stroke can induce gut barrier permeability and dysfunction, which further promote the translocation and dissemination of gut microbiota to peripheral tissue, leading to post-stroke infections.	(Stanley et al., 2016)
Mouse MCAO	Healthy fecal transplatation with homeostatic gut microbiota before stroke can pevent ischemic damage.	(Winek et al., 2016)
Mouse MCAO	Anti-inflammatory atorvastatin can restore gut microbiota and repair gut barrier after stroke, which contributes to anti-inflammatory responses in stroke recovery.	(Zhang et al., 2021)
Mouse MCAO	Gut microbiota-derived SCFAs can improve stroke recovery and neurological outcomes	(Sadler et al., 2020)
Pig MCAO	Systmatic inflammation and dysbiosis of gut microbiota are observed in acute phase of stroke.	(Jeon et al., 2020)
Mouse photothrombotic stroke model	Lactulose repairs gut barrier injury and improves gut microbiota dysbiosis after stroke; lactulose can also improve post-stroke neurological outcomes.	(Yuan et al., 2021)
Clinical cohorts and mouse MCAO	Gut microbiota dysbiosis can be used as a index to predict stroke outcome in both animals and patients.	(Xia et al., 2019)
Clinical cohorts and mouse MCAO	In both patients and animals, rapid and dynamic gut dysbiosis were observed after stroke; Enterobacteriaceae in turn induces post-stroke inflammation and can serve as biomarker or therapeutic target.	(Xu et al., 2021)
Clinical cohorts and mouse study	Gut microbiota-derived TMAO promotes inflammation and predicts high risk of cardiovascular events in stroke patients, making it a promising biomarker of poor outcome and prognosis.	(Haghikia et al., 2018)

MCAO, middle cerebral artery occlusion; SCFA, short-chain fatty acids; TMAO, trimethylamine N-oxide.

## Gut Microbiota Regulates Key Management Aspects During Post-Stroke Recovery

In stroke patients, body temperature, blood glucose, blood pressure, oxygen and hydration status are the key parameters in post-stroke management (Bhalla et al., 2001). These parameters may change either upwards or downwards after stroke, and require careful balance to reach favorable outcome and prognosis (Bhalla et al., 2001). When stroke reshapes the population of gut microbiota, they might in turn modulate these physiological parameters *via* diverse molecular and cellular mechanisms according to the recent advances.

### Immune Response

Neuroinflammation is a hallmark of ischemic stroke. In acute phase of stroke, neuroinflammation is considered to be partially beneficial by scavenging damaged tissues and promoting neuroregeneration (Yong et al., 2019). However, it predominantly exacerbates brain injury from sub-acute to chronic phase, and elevates the risk of stroke occurrence and recurrence (Esenwa and Elkind, 2016). Post-stroke population of gut microbiota can modulate neuroinflammatory responses through various pathways, with either advantageous or disadvantageous aspects. In 2016, three independent studies have demonstrated that intestinal dysbiosis can regulate cytokines and T cell functions in different patterns, by which they eventually influence stroke outcome. In the first study, the dysbiosis of gut microbiota induced by stroke has been reported to increase pro-inflammatory cytokines IL-17 and IFN-γ in recipient germ-free mice after fecal transfer, and lead to unfavorable outcome in recipient mice (Singh et al., 2016). In another report, however, antibiotic–induced dysbiosis of gut microbiota promoted anti-inflammatory cytokines IL-10 from T_reg_ cells and simultaneously suppressed pro-inflammatory cytokine IL-17 from γδT cells, thereby improved stroke outcome (Benakis et al., 2016). Interestingly, in the third study, gut microbiota depletion by antibiotics before injury can dramatically reduce survival rate after stroke, whereas either continuous subsequent antibiotic treatment or recolonization of gut microbiota can both increase animal survival (Winek et al., 2016). Regardless of stroke outcome, antibiotic-induced microbiota depletion suppressed B cells and several subtypes of T cells, resulting in a general immunodepression (Winek et al., 2016). Considering the diversity and the quantity of major populations in gut microbiota were both significantly reduced in these depletion models, the opposite effects indicate the complexity and uncertainty how gut microbiota influences stroke outcome. Moreover, a recent study has revealed that, the fecal transplants from severe stroke patients can enhance IL17 positive γδT cell number in recipient mice (Xia et al., 2019), providing potential clinical evidences to support the conclusions from the first study (Singh et al., 2016). However, more detailed analysis on the microbial colonization and the impact of specific bacterial species are required in future studies before any medical translation.

Notably, the metabolic products of gut microbiota may play also important roles in post-stroke inflammatory responses. Short-chain fatty acids (SCFAs) are typically produced during gut bacteria fermentation, of which the major components consists of acetate, propionate and butyrate (Sun et al., 2018). Gut microbiota-derived SCFAs can dramatically stimulate IL-10 production from Th1 cells (Sun et al., 2018), which has been widely reported as a key factor in favorable stroke outcome (Garcia et al., 2017). The similar effects of gut microbiota metabolites on IL-10 are also consistent with the findings in the second report (Benakis et al., 2016). A recent report has revealed that gut microbiota-derived SCFAs can significantly alter contralesional cortex connectivity thereby improve neurological outcome in mouse stroke model (Sadler et al., 2020).

In addition, gut microbiota may also modulate the risk factors resulting in primary and secondary stroke. For instance, gram negative bacteria in gut can activate TLR-4 in brain endothelial cells, thereafter drives cerebral cavernous malformations *via* TLR4-MEKK3-KLF2/4 pathway and significantly increases the occurrence and recurrence risk of stroke (Tang et al., 2017).

To sum up, it is believed that gut microbiota can regulate post-stroke immune responses through their byproducts and pathogen associated molecular patterns (PAMPs) to modulate immune cells and inflammation-related cytokine. However, it is still controversial whether gut microbiota drives immune responses to pro-inflammatory direction or anti-inflammatory direction. The factors and contexts determining their immune-modulatory functions remain largely unknown and require to be unraveled before applying gut microbiota-based therapy to clinical use.

### Body Temperature

Fever is a common symptom after ischemic stroke and usually associated with elevated mortality and morbidity (Saini et al., 2009). The mechanisms of hyperthermia-induced poor clinical outcome and prognosis involve injuries on intestinal barrier, increased pro-inflammatory cytokines and dysfunctions of blood-brain barrier (Zaremba, 2004; Oliver et al., 2012). Firstly, hyperthermia is widely known to disrupt intestinal mucosa barrier (Oliver et al., 2012), which leads to substantial changes in the community of gut microbiota (Stanley et al., 2018). The dysbiosis of gut microbiota can contribute to complex inflammatory responses as discussed above. Also, the dysfunction of intestinal barrier may lead to the translocation of gut microbiota to other organs, which increases the risk of systematic infection during hospitalization within first weeks after stroke (Fugate et al., 2014; Stanley et al., 2016). Furthermore, gut microbiota is indispensable to maintain the permeability of blood-brain barrier (BBB) by elevating the expression levels of tight junction proteins (Braniste et al., 2014). While BBB breakdown is exacerbated by hyperthermia and leads to a worsen outcome of ischemic stroke (Ginsberg and Busto, 1998), gut microbiota may provide beneficial effects in post-stroke recovery by maintaining the integrity of BBB. On the other hand, however, the thermoregulation function of gut microbiota is highly dependent on UCP1 signaling in brown adipose tissue (Li et al., 2019). The depletion of gut microbiota after antibiotic treatment could blunt toxin-induced hyperthermia by regulating UCP1 and TGR5 gene expressions in brown adipose tissue and skeletal muscle (Ridge et al., 2019), implying a positive effect of gut microbiota on hyperthermia.

In contrast to hyperthermia, slightly decreased body temperature predicts good functional outcome after stroke (Jorgensen et al., 1999). In fact, therapeutic hypothermia is one of the most encouraging methods in neuroprotection after acute brain injury including ischemic stroke (Wu and Grotta, 2013). The efficacy of hypothermia has been successfully validated in pre-clinical animal models, while its limitations remain to be solved in clinical trials (Wu and Grotta, 2013). The natural hypothermia, namely hibernation, has been demonstrated to alter the major populations in gut microbiota, including *Bacteroidetes*, *Firmicutes, Verrucomicrobia*, *Deferribacteres*, *Cyanobacteria* and *Actinobacteria* (Chevalier et al., 2015). Conversely, cold-responding microbiota orchestrates energy homeostasis by modulating insulin sensitivity (Chevalier et al., 2015), which is one of the key concerns during stroke recovery (see below, the section of ‘blood glucose’). In addition, microbiota-depletion mice also showed impaired UCP1-dependent thermogenesis upon cold exposure (Li et al., 2019). However, clinical evidence remain insufficient to confirm the link between hypothermic-induced gut microbiota alteration and stroke outcome. Only one clinical study analyzed gut microbiota composition in therapeutic hypothermia-treated patients after hypoxic-ischemic encephalopathy (HIE) (Watkins et al., 2017), a similar ischemic brain injury occurring mainly in neonates. As reported by Watkin’s *et al.*, the diversity in global microbial richness and the proportion of *Bacteroidetes* were both significantly reduced in hypothermia-treated HIE patients when comparing to healthy individuals (Watkins et al., 2017).

To summarize, the population of gut microbiota is significantly altered when body temperature changes. Hyperthermia-induced dysbiosis may exacerbate stroke severity, whereas hypothermia-induced dysbiosis may ameliorate post-stroke symptoms, in consistent with the clinical management strategies of body temperature in stroke patients. In addition, gut microbiota may in turn regulate body temperature through UCP1 signaling in brown adipose tissue, which provides a novel insight for body temperature management after stroke.

### Blood Glucose

The dysregulation of blood glucose level is a common post-stroke symptom. Both hyperglycemia and hypoglycemia can result in detrimental brain damage and lead to a unfavorable clinical outcome (Lindsberg and Roine, 2004; Dave et al., 2011). In particular, diabetic patients often suffer a higher risk and poorer outcome and prognosis of ischemic stroke than non-diabetic population, while a high proportion of stroke patients also bear hyperglycemia even without diabetic history (Luitse et al., 2012). Large-scale sequencings have revealed the dysbiosis patterns of gut microbiota in type-II diabetic patients, including the reduced abundance of some butyrate-producing bacteria (Qin et al., 2012; Karlsson et al., 2013), which may result in decreased insulin sensitivity (Gao et al., 2009). Moreover, the dysbiosis of gut microbiota may lead to the dysregulation of CD4 T cell homeostasis and induce glucose intolerance as well as insulin resistance (Garidou et al., 2015). In contrast, transplantation of gut microbiota from healthy donors can increase insulin sensitivity in patients with metabolic dysregulation (Vrieze et al., 2012). Additionally, microbiota-derived SCFAs have been suggested to maintain cardiovascular health by several mechanisms including the regulation of glucose homeostasis (Chambers et al., 2018). Their effects on cardiovascular functions may contribute to a reduced risk of stroke occurrence and recurrence. Based on current findings, healthy gut microbiota may assist the homeostasis of blood glucose, which favors stroke recovery and prognosis. Interestingly, the role of gut microbiota in glucose homeostasis is independent from their role in adaptive thermogenesis (Krisko et al., 2020), indicating that we may uncouple their functions in regulating body temperature and blood glucose during post-stroke management to meet individual therapeutic demands.

### Blood Pressure

Similar to blood glucose, blood pressure is also commonly dysregulated and associated with adverse prognosis after ischemic stroke (Potter et al., 2009). Both hypertension and hypotension require immediate intervention to drive blood pressure to normal level (Potter et al., 2009). Excessive salt intake (> 5g/day) is one of the main risk factors of stroke by elevating blood pressure (Li et al., 2018b). Therefore, low-sodium dietetic control is recommended in post-stroke management, which favors recovery and reduces the risk of stroke recurrence. Recently, specific species of gut microbiota such as *Lactobacillus murinus* have been identified to prevent salt-sensitive hypertension by regulating T_H_17 cell functions (Wilck et al., 2017). A clinical report supports the conclusion by showing certain *Lactobacillus* species are negatively associated with sodium intake and blood pressure, although the overall gut taxonomic composition showing no obvious correlation (Palmu et al., 2020). Furthermore, gut microbiota-producing metabolites like SCFAs and trimethylamine N-oxide (TMAO) are also highly involved in the regulation of blood pressure (Marques et al., 2018). While SCFAs tend to exert protective functions to avoid hypertensive cardiovascular injury (Bartolomaeus et al., 2019), TMAO on the other hand promotes hypertension and results in atherosclerosis and cardiovascular diseases (Wang et al., 2011; Wang et al., 2015). Actually, increased TMAO production has been considered as a potential biomarker to predict high risk of cardiovascular diseases (Nam, 2019). The changes of gut microbiota community are also observed in arteriosclerotic patients (Karlsson et al., 2012). To our current knowledge, only specific bacteria species in gut microbiota can regulate blood pressure by immunomodulation and metabolite-induced effects. Species like *Lactobacillus* may serve as a therapeutic target to control post-stroke blood pressure. While the effects of diverse metabolites may drive the disease progress in opposite directions, therefore accurate analysis on specific species and their metabolic products are required to deepen our understanding on gut microbiota and blood pressure control after stroke.

### Oxygen

Insufficient oxygen supply (hypoxia) occurs in ischemic stroke due to the blockage of cerebral blood flow and the lack of oxygen store in brain, which results in subsequent neurological deficiency (Ferdinand and Roffe, 2016). However, the efficacy and safety of oxygen supplementation, which leads to hyperoxia, have also become controversial based on the results of recent clinical trials (Rincon et al., 2014; Roffe et al., 2017). The control of oxygen level in stroke patients remains as a dilemma. As commensal gut microbiota physiologically reside in a hypoxic to anoxic environment, the elevation of oxygen concentration in both mouse and human can break the homeostasis of gut microbiota and lead to dysbiosis (Albenberg et al., 2014), which could have a strong impact on stroke as discussed above. In hyperbaric oxygen therapy, hyperoxia may also induce dysbiosis of gut microbiota through matrix metalloproteinease-9 (MMP-9) (Cummins and Gentene, 2010; Rodrigues et al., 2012). Until now, only a few studies are involved in the mechanisms how gut microbiota influences oxygen level. In insect, gut bacteria can reduce gut oxygen level as a signal for larvae development (Coon et al., 2017). In rodent model, gut microbiota-producing butyrate activates PPAR-γ signaling pathway and facilitates nitrate production, through which oxygen is excluded outside of the colon lumen and prevents dysbiosis in gut ecosystem (Byndloss et al., 2017). Notably, depletion of butyrate-producing *Clostridia* in gut microbiota can also lead to an aerobic luminal expansion (Rivera-Chavez et al., 2016). As a typical translocating species after stroke (Stanley et al., 2018), *Clostridia* may also modulate oxygen microenvironment in peripheral tissues and organs. A recent study has revealed that lung and gut microbiota communities can be significantly altered by hyperoxia (Ashley et al., 2020). Gut microbiota can correlate with lung inflammation and in turn protect lung from oxygen-induced injury (Ashley et al., 2020), which may indicate certain functions of translocated gut microbiota after stroke (Stanley et al., 2016). However, more evidence are demanded for the precise interpretation of the crosstalk between gut ecosystem and systematic and localized oxygen modulation during post-stroke management.

### Hydration

Water balance is also highly concerned in the management of stroke recovery. In elderly stroke patients, both overhydration and dehydration status exist (O’Neill et al., 1992). Dehydration is of high frequency and particularly associated with poor outcome and prognosis of stroke (Rowat et al., 2012). Secreted chloride anion (Cl^-^) from gut epithelial cells facilitates water transport and hydration, in which the composition of gut microbiota is significantly shifted (Keely et al., 2012; Musch et al., 2013). Unfortunately, little data is available on whether gut microbiota reversely affects hydration status. In general, investigations on how gut microbiota regulates osmolality are still very limited, thus more studies need to be performed not only to describe phenotypes but also to elucidate underlying mechanisms before we can take advantage of gut microbiota in hydration homeostasis in post-stroke management.

## Conclusion

**Figure 1:**
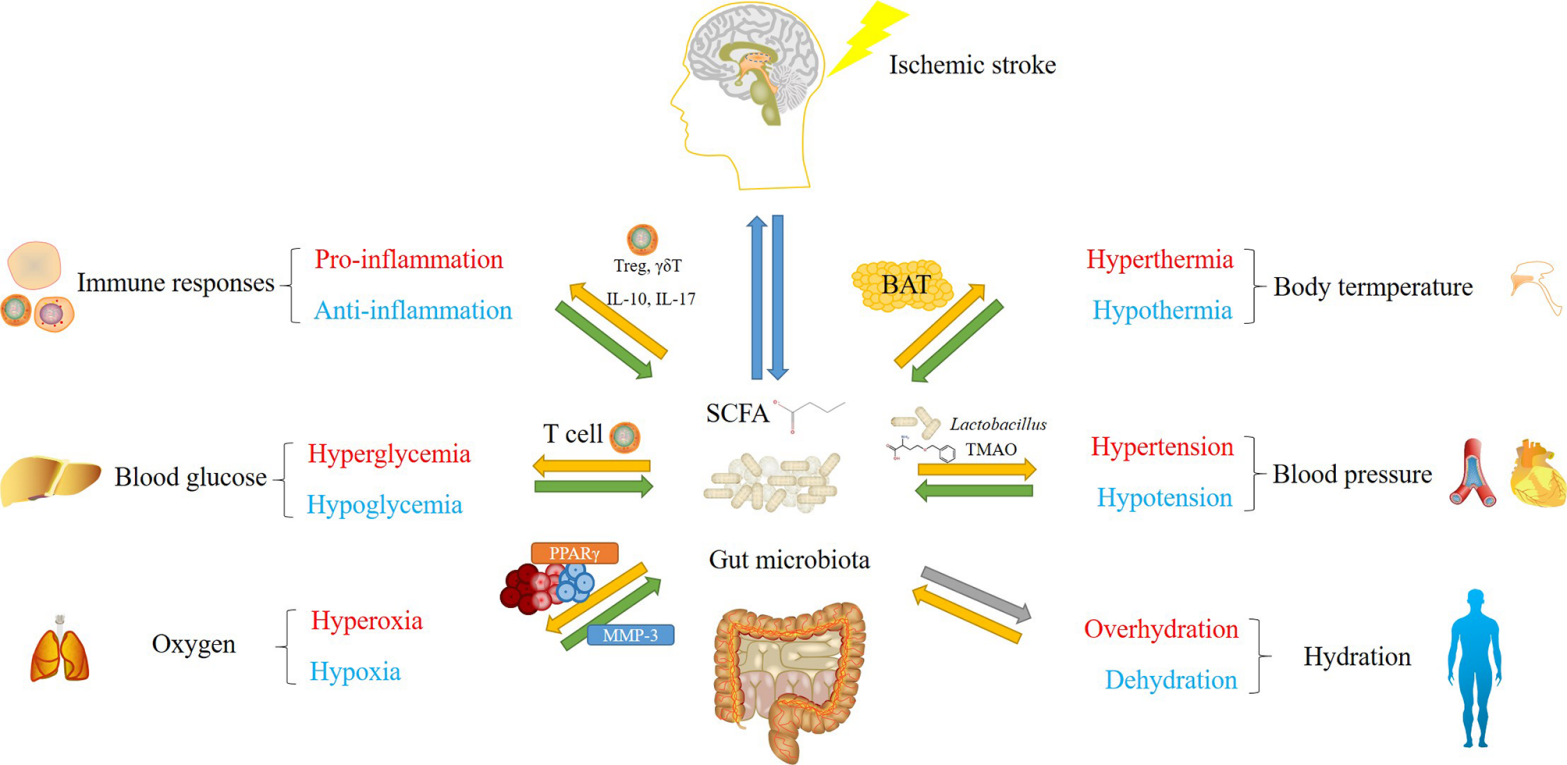
An overview of major concerns in post-stroke management influenced by gut microbiota. Green arrows represent solid functions, yellow arrows represent opposite functions or functions with relatively weaker evidence, gray arrows show effects without solid evidence. Gut microbiota and their metabolic products, mainly short-chain fatty acid (SCFA), regulate immune responses, body temperature, blood glucose, blood pressure and oxygen level after stroke. Their populations and diversities are also altered by these factors. Gut microbiota-mediated modulation involve mechanisms including the regulation of T cell (immune responses and blood glucose), the regulation of UCP1 gene expression in brown adipose tissue (BAT) (body temperature), SCFA and trimethylamine N-oxide (TMAO)-mediated opposite effects on blood pressure, and PPARγ-mediated oxygen exclusion. Although gut microbiota populations are considered to be influenced by altered hydration status, whether they conversely regulate hydration remains unclear.

Accumulating evidence support a crucial role of commensal gut microbiota in stroke prevention and recovery management. Notably, gut microbiota and their metabolites could be involved in post-stroke regulation of immune responses, body temperature, blood glucose and blood pressure in both directions, while it remains unclear whether gut microbiota could regulate oxygen and hydration levels after stroke (**Figure 1**). Considering the complexity of the populations and metabolites in gut microbiota, their effects on ischemic stroke could be highly variable, making it difficult to apply gut microbiota directly to stroke recovery. Therefore, instead of using fecal transplantation containing entire gut microbiota populations from healthy donors, discovering specific functional bacteria species or metabolic compounds from homeostatic gut microbiota could be a wise strategy to promote stroke recovery in an efficient and safe manner. Actually, researchers have already attempted to develop drugs modulating gut microbiota in the stroke therapy using rodent models. For instance, *Panax Notoginsenoside* extract has been shown to protect rat brain from ischemic stroke by regulating GABA-β receptors *via* the modulation of gut microbiota populations, particularly through *Bifidobacterium longum* (Li et al., 2018a). Interestingly, oral administration of *Bifidobacterium longum* has achieved similar neuroprotective effects as *Panax Notoginsenoside* extract (Li et al., 2018a), which serves as an example how we could take advantage of specific species from gut microbiota for stroke recovery. In addition, atorvastatin and lactulose have already been demostrated to repair gut barrier, reduce gut inflammation and restore gut microbiota after stroke, providing novel startegies to improve stroke outcome (Yuan et al., 2021; Zhang et al., 2021). In the future, both basic research and clinical studies on gut microbiota will open novel avenues not only for stroke therapy, but also for other brain disorders.

## Author Contributions

XZ conceived the idea and wrote the manuscript. JZ, SL, and JY assisted with reference collection, figure and table preparation. All authors contributed to the article and approved the submitted version.

## Funding

This work was supported by the Foundation of Shenzhen-Hong Kong Institute of Brain Science-Shenzhen Fundamental Research Institutions-Shenzhen Fundamental Research Institutions (NSY889021031) and the Start-up Fund of Shenzhen Institute of Advanced Technology, Chinese Academy of Sciences (E1G0241001).

## Conflict of Interest

The authors declare that the research was conducted in the absence of any commercial or financial relationships that could be construed as a potential conflict of interest.

## Publisher’s Note

All claims expressed in this article are solely those of the authors and do not necessarily represent those of their affiliated organizations, or those of the publisher, the editors and the reviewers. Any product that may be evaluated in this article, or claim that may be made by its manufacturer, is not guaranteed or endorsed by the publisher.
